# Sensors and Techniques for On-Line Determination of Cell Viability in Bioprocess Monitoring

**DOI:** 10.3390/bioengineering9120762

**Published:** 2022-12-03

**Authors:** Laura S. Rösner, Franziska Walter, Christian Ude, Gernot T. John, Sascha Beutel

**Affiliations:** 1 Institute for Technical Chemistry, Leibniz University of Hanover, 30167 Hannover, Germany; 2 PreSens Precision Sensing GmbH, Am BioPark 11, 93053 Regensburg, Germany

**Keywords:** sensors, spectroscopy, viability, cell culture, bioprocess, monitoring, soft sensor

## Abstract

In recent years, the bioprocessing industry has experienced significant growth and is increasingly emerging as an important economic sector. Here, efficient process management and constant control of cellular growth are essential. Good product quality and yield can only be guaranteed with high cell density and high viability. Whereas the on-line measurement of physical and chemical process parameters has been common practice for many years, the on-line determination of viability remains a challenge and few commercial on-line measurement methods have been developed to date for determining viability in industrial bioprocesses. Thus, numerous studies have recently been conducted to develop sensors for on-line viability estimation, especially in the field of optical spectroscopic sensors, which will be the focus of this review. Spectroscopic sensors are versatile, on-line and mostly non-invasive. Especially in combination with bioinformatic data analysis, they offer great potential for industrial application. Known as soft sensors, they usually enable simultaneous estimation of multiple biological variables besides viability to be obtained from the same set of measurement data. However, the majority of the presented sensors are still in the research stage, and only a few are already commercially available.

## 1. Introduction

The most frequently measured biological parameters of industrial biotechnological processes are cell density and the viability of cultivated cells. Measurement of these key parameters provides the basis for any decision regarding process control, e.g., feeding rate, time of induction and transfection or harvesting [1]. 

In order to operate biotechnological processes efficiently, the selection of an appropriate method for determining viable cell density is crucial. To date, off-line sensing techniques have been the most popular techniques for monitoring the cell viability of cultivated microorganisms and mammalian cells. Nevertheless, off-line methods often require time-consuming sample preparation, and sometimes, long incubation times impede real-time determination. Due to the time-delayed output of results, off-line methods are not well suited to decision making in process regulation and control [2]. Furthermore, they can only provide a very selective statement of cell viability throughout the entire process and do not supply any information about the current trend of a cultivation.

On-line sensing techniques have numerous advantages compared to off-line methods, minimizing measurement delay and providing a continuous flow of information about the state of the cultivation system [3]. Thus, ongoing processes can be controlled and intervened with in real-time, and even short-term changes in cell metabolism due to diauxic growth, the Crabtree effect or substrate limitation can be visualized [4].

On-line measurement of important physical process parameters such as pH, temperature or dissolved oxygen is common practice in industrial biotechnology. On-line measurement of cell viability is not yet well established due to the lack of versatile, robust, non-invasive sensors and the requirement of extensive bioinformatics data processing, and has so far been limited to special cases only. To determine cell viability, only capacitance probes are commercially available as on-line sensors [5,6,7]. However, the determination of total cell density and, in particular, the viable cell density of suspension cell cultures plays an outstanding role in upstream processing in the biopharmaceutical industry. In recent years, the number of commercialized tools for the on-line measurement of these two parameters has increased steadily, not least due to the strict requirements in quality management regarding the certification of the biotechnological production process (QbD—quality by design) [2].

Another key driver for the rapid development and improvement of on-line measuring methods has been the “Process Analytical Technology” (PAT) initiative of the US Food and Drug Administration (FDA), which aims to achieve good product quality through early failure detection [8]. To fulfill the demands of this initiative, continuous monitoring of the critical process parameters (CPP) is necessary, which requires powerful on-line sensing methods [1].

In this review, several applications of the following spectroscopic sensing principles in monitoring viability in biotechnological processes will be discussed:

- UV-vis spectroscopy
- Fluorescence spectroscopy
- Infrared spectroscopy
- Raman spectroscopy
- Dielectric spectroscopy

The majority of the measurement principles presented in this review are spectroscopic on-line methods. Some of the examples displayed can currently only be applied off-line. With appropriate further development, however, these techniques could also be implemented as on-line methods if developed further.

## 2. Basic Principles of Bioprocess Monitoring and Viability Determination

Bioprocesses involve multiple steps, including upstream processing, downstream processing and product formulation. Each of these steps must be monitored and regulated precisely, which requires suitable sensors that meet specific demands. Both the process itself and the process monitoring can be arranged differently. Likewise, this applies to the determination of viability, with each method possessing advantages and disadvantages.

### 2.1. Bioreactor Modes of Operation and Monitoring Techniques

Often, productivity, i.e., the amount of a product that can be obtained per volume and per time, is considered almost more important than yield in industrial bioprocesses [9]. In order to achieve optimum productivity, the bioprocess and the type of reactor must be well matched and the process has to be precisely regulated and controlled. Depending on the design and operation of the reactor, different reactor types can be distinguished. The stirred tank reactor (STR) is one of the most widely used reactors, and can be operated not only as a batch reactor but also continuously as a continuous stirred tank reactor (CSTR) [9]. 

In principle, a bioreactor can be operated in three different ways: batch, fed-batch and continuous [10].

In a batch process, a known initial concentration of cells and substrates is used without adding further media during the process [11,12]. Only after the cultivation is complete are the products removed and purified. Hence, no input or output of liquids occurs and the liquid volume in the vessel can be considered constant [10].

Especially in industrial production, fed-batch processes are very common. Such processes are usually the method of choice when higher initial substrate concentrations are not applicable due to inhibitory (catabolite repression) or even toxic effects. Fresh medium or substrate is added intermittently or continuously during fed-batch processes. Thus, a free volume must remain in the reactor for the added medium at the beginning of the cultivation. By using a fed-batch process, the frequency of downtime that occurs in the batch processes can be reduced, which often makes fed-batch processes a more economical alternative compared to batch processes [12].

In continuous processes, the substrate is continuously fed via the feed stream and products are removed via the product stream. Depending on the method of maintaining the steady state, a distinction can be made between chemostats and turbidostats [10]. To increase the productivity of a CSTR, cell retention can be implemented. Reactors using cell retention are referred to as perfusion bioreactors [9]. Another reactor type, although only occasionally used in biotechnology, e.g., when valuable gaseous substrates are involved, is the plug flow reactor (PFR) [9].

In particular, fed-batch and continuous processes require precise regulation to ensure optimal process control. The classification of sensor types for process control is based on their position in the process and the method and frequency with which they provide information on the process. A basic distinction is made between on-line, at-line and off-line sensors.

During off-line analysis, individual samples are taken from the reactor and examined in an (external) analytical laboratory [12]. The resulting time delay often impedes efficient process control, since the window for intervention in the process has usually elapsed by the time the result is available [12]. Additionally, whether sampling is performed automatically (at-line) or manually (off-line) is often associated with a high risk of contamination. Hence, on-line sensors are preferable [2,13,14].

As mentioned above, at-line sensors require regular (automatic) sampling by means of a suitable sampling device. This procedure is often used for analysis via chromatographic methods or mass spectrometry (MS). Biosensors are usually operated at-line in flow injection analysis (FIA) systems as they must remain outside the sterility barrier [15]. Although this method involves a time delay, it has the advantage that samples can be adjusted to the optimal assay conditions.

On-line sensors, in contrast, are directly in contact with the bioprocess and are either located directly inside the reactor (invasive) or separated from the reactor by the reactor wall (non-invasive) [12]. They can also be operated in bypass mode, with a stream continuously diverted from the reactor and measured in flow-through mode. On-line sensors can deliver results directly from the reactor environment in real time without the need for manual interaction [13,15,16].

### 2.2. Sensor Requirements

Devices considered for on-line-monitoring are required to possess certain attributes which qualify them for their use in bioprocess monitoring and control.

In order to endure the harsh conditions during sterilization, the sensor, or at least the optical window for the sensor, must be robust enough and needs to maintain its calibrated state [13,16]. In addition, the sensor should not interfere with the sterile barrier [16]. The measurement accuracy of the sensor is determined by various interactions with the bioreactor and the cultured species. If increased cell debris occurs, as is the case in the cell death phase, spectroscopic analysis of the culture broth can become more complicated. In addition, the measurement can be affected by gas bubbles, solid particles, stirring and very high cell densities. For these reasons, a consistently good signal-to-noise ratio is crucial for applicability of the sensor throughout the entire cultivation process, regardless of changes in chemical and physical process parameters. 

Viability sensors must also fulfill general sensor criteria, including high specificity and high selectivity. While a sensor’s selectivity represents its ability to measure a target analyte in presence of other compounds, sensitivity refers to the change in the output signal as a result of a change in analyte concentration [17]. Other criteria such as stability, linearity, robustness and repeatability must also be fulfilled [2].

Sensor requirements also depend on the cultured species, the type of medium and reactor, as well as the achieved cell density. While the cultivation of mammalian cells requires very sensitive and specific sensors with low detection limits, microbial production processes with high occurring cell densities, high viscosity and high gassing rates impose very high demands on the robustness of the sensors used for monitoring [8].

Especially in the field of single-use (SU) bioreactors, there is a particularly great need for the research and development of viability sensors [18]. Invasive probes which are sterilized within the reactor are usually not well suited to single-use application as they cannot always reliably guarantee the integrity of the sterile barrier. Non-invasive spectroscopic methods, on the other hand, can be easily implemented in SU bioreactors, which also prevents the risk of cross-contamination [19]. However, one challenge which must be overcome for sensor application in SU bioreactors is the permeability of the plastic reactor material for electromagnetic waves, to enable measurement in the reactor bag without loss of intensity or interference effects. In addition, the development of plug-and-play devices for sensor integration in SU devices is of particular interest [20]. One reason for this effort is that standardized ports such as the Ingold port are not available for SU applications [17].

### 2.3. Off-Line Methods for Viability Determination

Even though on-line sensors offer promising possibilities for the determination of viability, they have not been widely used to date. Off-line methods are still the method of choice and are stipulated in many standard operating procedures (SOPs) for quality management.

The choice of the appropriate test method depends on the cultured organism. The standard method for the assessment of bacterial viability is the colony count method. However, this method requires several days of incubation for colony formation and is limited to culturable bacteria that grow on agar plates. Furthermore, it can be difficult to obtain reproducible results due to the high sensitivity of the test to changes in the culture conditions and human counting errors [21].

A more robust off-line method than traditional cell counting is cell viability assays. The most prominent amongst these assays are dye exclusion assays, colorimetric assays, luminometric assays and flow cytometric assays [22]. Nevertheless, a disadvantage of these viability assays is the requirement of several (time-consuming) steps for preparation and analysis and the use of detection devices such as (fluorescence) microplate readers, (fluorescence) microscopes or flow cytometers.

According to not only commercial GMP manufacturing but also to research and process development, the gold standard for the simultaneous determination of total cell density and viable cell density is at-line analysis via live/dead staining [1]. These so-called dye exclusion assays, e.g., trypan blue, propidium iodide or 7-aminoactinomycin D (7-AAD), are based on the membrane integrity, and the dyes can enter the cells when cell death occurs [23]. The counterstaining of living cells can be performed using calcein acetoxymethyl (calcein AM) [22].

A variety of tetrazolium compounds can also be used to detect viable cells. 3-(4,5-dimethylthiazol-2-yl)-2,5-diphenyltetrazolium bromide (MTT) is positively charged and can penetrate viable eukaryotic cells [24]. MTT is reduced to a purple formazan product by cells with an active metabolism [25]. This reduction can be quantified by measuring absorbance at 570 nm after one to four hours of incubation. It was one of the first viability assays suitable for high-throughput screening. The assay has to be considered as an endpoint assay, as MTT has a cytotoxic effect and can be influenced by reducing compounds in the medium such as ascorbic acid or coenzyme A [24].

The resazurin reduction assay is comparable to the MTT-assay. Similar to MTT, resazurin also acts as a redox indicator. Viable, metabolically active cells can reduce resazurin, leading to the pink, fluorescent resorufin. However, the MTT-assay has limitations as well, since media compounds may interfere with the fluorescence of resorufin which, itself, is cytotoxic [24,25].

In contrast, adenosine triphosphate (ATP)-based assays function differently. Here, the addition of the assay reagent leads to immediate rupture of the cell membrane; therefore, no incubation is required [24]. This assay utilizes the enzyme firefly luciferase, which converts luciferin, resulting in a long-lasting luminescent signal [25]. It provides a very rapid, sensitive method to determine viability and takes advantage of the fact that cell death is accompanied by loss of membrane integrity. However, a transmembrane proton gradient is mandatory for ATP synthesis. Consequently, ATP synthesis becomes impossible after cell death and any remaining ATP in the cytoplasm is consumed by endogenous ATPases [24].

Flow cytometry as a method of quantitative single-cell analysis can be used for viability determination as well. With this method, cells can be characterized within a liquid flow with the aid of lasers, depending on their size, granularity or ability to carry specific fluorescent molecules. Since dying cells are often smaller than viable cells, changes in viability can be observed via forward- and side-scatter analysis. Common cytotoxicity and viability staining methods can also be applied to cytometry [22].

## 3. Spectroscopy-Based Techniques for On-Line Monitoring of Viability

**Figure 1:**
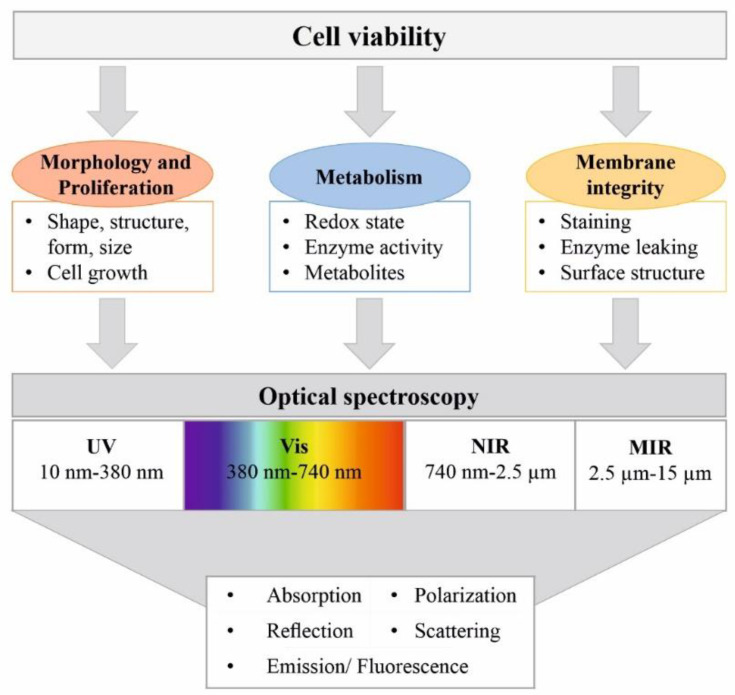
Application of spectroscopic methods for cell viability determination. Changes in cell morphology, metabolism and membrane integrity due to a change in cell viability can be detected via spectroscopic methods. Various optical properties in specific wavelength ranges can be exploited for this purpose.

Spectroscopic sensors offer an effective method of continuous process monitoring and are capable of providing detailed information on the molecular state of the process, as well as on the physical and metabolic state of the cells. Because there are no reagents, no sampling is needed and no analyte consumption occurs, on-line sensors and spectroscopic measurement methods show great potential as non-invasive in situ techniques for viability determination. Since optical systems do not interfere with metabolism, in vivo measurements are feasible and they can even be utilized to yield intracellular information [26]. All spectroscopic methods rely on the interaction of electromagnetic waves with molecules or matter, which results in a specific electromagnetic spectrum. This enables the investigation of changes in the chemical composition or the physical structure of the probed cells that occur during cell death, as shown in Figure 1 [27].

Since spectroscopic methods are usually not focused on single components but provide large sets of data, relevant data need to be extracted and mathematical procedures for data mining are mandatory [27].

While capacitance probes fitting common bioreactor ports are commercially available, the connection of optical sensors to the bioreactor is more difficult. Fiber-optic waveguides are commonly used to transmit light [28]. Cell culture analysis can also be performed using an optical window or a flow cell, which allows the light to penetrate the culture broth [29]. 

### 3.1. UV-Vis Spectroscopy

**Figure 2:**
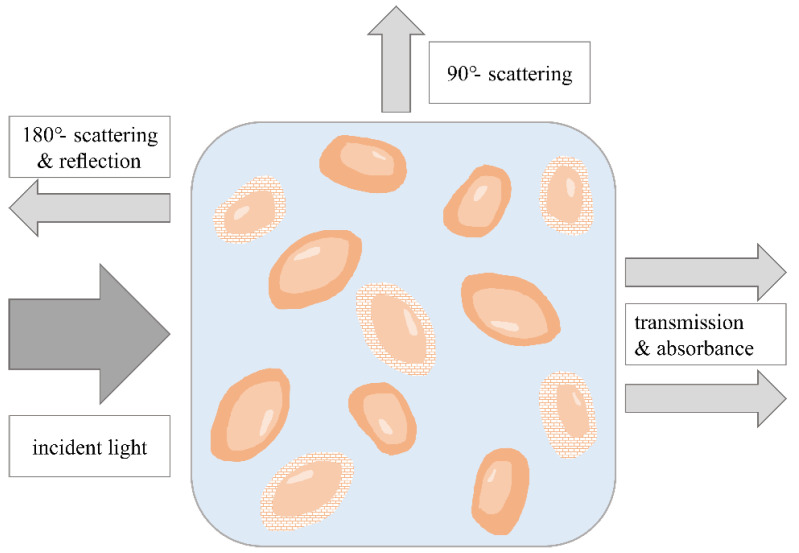
Monitoring of cell viability using different UV-vis spectroscopic methods. Different physical effects can be used for analyzing turbid cell suspensions depending on the arrangement of the light source and the detector. Based on [2].

Ultraviolet–visible (UV-vis) spectroscopy analyzes the interaction between a sample material and radiation in the wavelength range of 200 nm to 740 nm. It includes different physical effects, e.g., absorption, scattering, diffraction, refraction and reflection, and is best suited to the detection of chemical functions associated with lower-energy electronic levels (e.g., multiple bonds and aromatic groups) (Figure 2). Saturated hydrocarbons or sugars cannot be detected [28].

#### 3.1.1. Absorption Measurements

Devices for absorption measurement typically consist of a light source focused on the measuring chamber (cuvette or flow cell). Only photons that match the energy gap of the sample molecules are absorbed and excite the target molecules. The remaining photons transmit the measuring chamber and can be detected on the opposite side. An absorption spectrum is obtained via stepwise comparison of the incident light intensity at a certain wavelength with the transmitted light at this wavelength [2]. 

UV-vis spectroscopy is one of the standard methods used to estimate the number of cells in a solution, as well as for the quantitative determination of substrates, metabolites or other compounds in the culture broth such as proteins or nucleic acids [13]. Although it has already been routinely used in a broad range of applications in biotechnology for many years, its use in viability testing is not yet as popular as its use for biomass determination.

Park et al. analyzed the normalized optical densities (OD) between 200–290 nm in *Escherichia coli*, *Bacillus subtilis* and *Staphylococcus epidermis*. Samples containing a higher proportion of living bacteria showed higher optical densities than samples with a higher number of dead cells. According to Park et al. these results can be explained by the different quantities of intracellular materials present in living and dead cells, e.g., nucleic acids. In addition, the ratio of OD, measured at 230 nm, to OD, measured at 670 nm, has been evaluated with respect to bacterial viability. The results showed a linear correlation with R^2^ values of 0.9964 to 0.9118 depending on the analyzed organism. The proposed method has also been successfully utilized for determining bacterial viability in bioaerosols [30]. 

Drieschner et al. assessed the critical process parameters of cell density and viability during the cultivation of Chinese hamster ovary cells (CHO) by mimicking the exponential phase and cell death phase via an inverse cultivation protocol. Here, different proportions of viable and dead cells were obtained by mixing fresh media, a cell broth only containing dead cells and a living culture at corresponding proportions. Although differences could be observed in the absorbance spectra of dead and viable cells, especially around 260 nm and 360 nm, no spectroscopic feature clearly contributed to cell viability. For this reason, further data analysis was executed using partial least square regression (PLS) and the extraction of cell viability from the obtained spectra was carried out using a multivariate curve resolution (MCR) model. Application of the model to two independent datasets showed a high correlation and low errors, and therefore, a good quality of external prediction (R^2^ = 0.984 for viable cell density and R^2^ = 0.993 for dead cell density) [31].

The capability of absorption spectroscopy in the range of 300–900 nm to characterize *E. coli* suspensions exposed to different inactivation methods was investigated by Kiefer et al. Their experiments revealed a correlation between the change in absorption in defined wavelength ranges and cellular damage. While a spectral range around 420 nm enabled the sensitive determination of cell density, changes in the range of 350–400 nm indicated damaged cell membranes. Substances that leaked out of damaged cells could be detected in the range between 800–900 nm [32]. UV-vis absorption spectroscopy combined with PLS and principal component analysis (PCA) has also been used to test the antibiotic resistance of *E. coli* and to monitor fecal indicator bacteria in water via determination of the cellular state [33,34].

Even organisms with a high concentration of cellular pigments, such as cyanobacteria, can be analyzed using absorption spectroscopy. By measuring absorption in the range of 300–800 nm and combining the obtained results with results from OD measurements at 750 nm (OD750) and fluorescence microscopy, the viability of *Synechocystis* sp. PCC 6803 was successfully determined. Both OD_750_ and the height of the peaks of phycocyanin, chlorophyll and carotenoids in the absorption spectra decreased with the loss of viability [35].

#### 3.1.2. Light-Scattering Measurements

Along with measurement techniques based on the analysis of turbidity, light-scattering methods are an alternative option to plate counting as well, and can be used to discriminate between live and dead cells [36,37]. Other than that, light scattering has long been used to characterize cell population parameters, the morphological properties of cells and even subcellular structures [38]. Thus, viability is also expected to influence cell properties with regard to light scattering [39].

In flow cytometry, forward- (FSC) and side-scatter intensity (90°, SSC) can be analyzed to obtain information on the size or the internal granularity of single cells [40,41]. However, angle-dependent scattered light measurements of whole cell populations are rather rarely performed. A disposable flow cell for cell density monitoring, based on infrared scattering measurements at five different angles with 180° backscattered light used for reference measurement, was developed by Raithel et al. [29]. Although this flow cell was not intended for viability determination, its design could be transformed and adapted to future viability determination. 

Cross and Latimer analyzed the angular dependence of light scattering from *E. coli* cells [42]. A reproducible characteristic scattering profile could be obtained for scattering angles between 20° and 90°. Furthermore, results indicated that microbial cells principally also fulfill main scattering theories such as the Rayleigh–Debye theory and Mie theory [42,43]. Technically, these scattering theories are limited to artificial spherical bodies, but in particular, the Rayleigh–Debye approximation for coated ellipsoids can be applied to explain the dependence of light scattering on membrane integrity, which could be exploited for viability determination [42].

Loske et al. described the utilization of dynamic light scattering (DLS) as a reliable, fast and easy method to determine the viability of *E. coli* in the lag and exponential phases using an analysis of growth rate for the calculation of viability [36]. DLS is based on inelastic scattering of the photons of a laser due to their interaction with suspended particles and allows both determination of the total scattered light intensity and analysis of the size of scattering particles. The light undergoes a frequency shift when it is diffracted by moving particles contained in the suspension. Using the Doppler effect, it is possible to determine the particle size if the temperature is kept constant [37]. Since the scattered light intensity is proportional to the concentration of the light-scattering particles, the slope of the scattered light intensity signal correlates directly with the growth rate of living bacteria acting as scattering particles. DLS usually uses visible laser light, with 90° being the most frequently measured scattering angle [44].

The application of dynamic light-scattering techniques is limited by the turbidity of the media. Sample vials have to be cleaned properly, as other (larger) particles highly influence scattering measurements [36]. Nevertheless, DLS is a highly adaptable technique which can be applied in situ under various physicochemical conditions [44].

### 3.2. Fluorescence Spectroscopy

**Figure 3:**
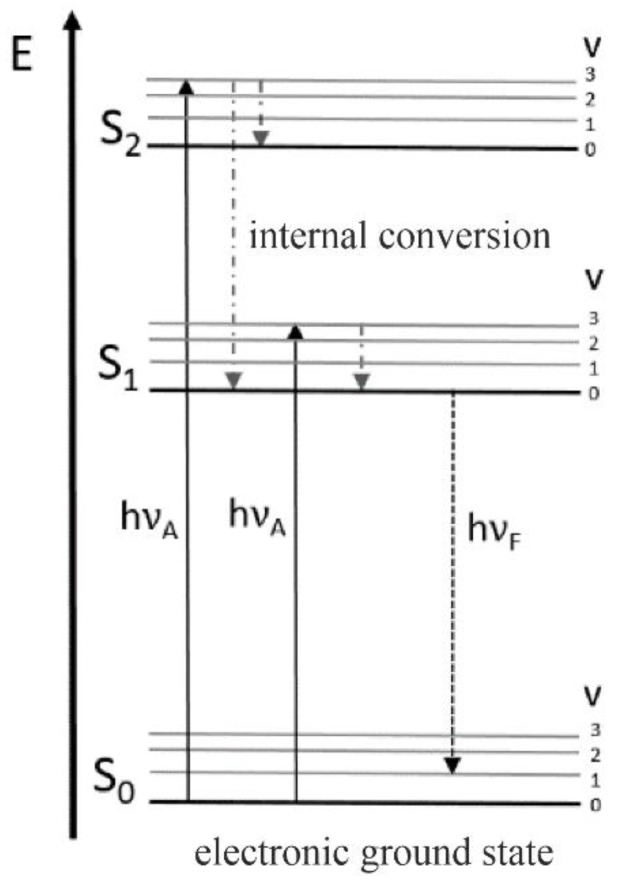
Jablonski diagram for illustration of electronic states and the radiative and non-radiative transitions between electronic states explaining fluorescence phenomena. Adapted with permission from [47]. Faassen and Hitzmann, 2016.

Another powerful tool for analyzing viability in bioprocess monitoring is fluorescence spectroscopy since living cells contain multiple endogenous fluorophores which are mainly involved in metabolic activity or cellular growth. Amongst these fluorophores, fluorescent amino acids (phenylalanine, tyrosine and tryptophan), cofactors (FAD, FMN, NADH and NADPH) porphyrins and vitamins (riboflavin) are the most significant ones when it comes to determining the viability of biological samples via fluorescence spectroscopy [2,45,46]. Based on the existence or absence and the amount of these substances, fluorescence spectroscopy offers a wide range of information on the cellular state, and thus, has been used for bioprocess monitoring for many years [47,48]. The physical mechanism of the fluorescence process is illustrated in Figure 3. 

When a fluorescent compound absorbs a photon, it reaches the excited state. Returning to the ground state involves the emission of a photon with a different frequency than the one causing excitation. Due to the occurrence of vibrational relaxation in the excited state, the emitted photon is of lower energy, causing a redshift in the emitted light [14,47]. 

However, fluorescence measurements can be influenced by different effects mainly involving changes in energy transfer and absorption. When non-fluorescent compounds of the culture broth absorb radiation, inner filter effects occur and fluorescence intensity can be reduced. Moreover, physical, chemical and biological process variables (e.g., pH, temperature, aeration, viscosity and optical density) can also affect fluorescence intensity since the electronic state that is responsible for the occurrence of fluorescence is specific to the ionization state of the fluorophore [47]. 

Multivariate data analysis is required to extract relevant information from fluorescence spectra. Since the information on cell viability included in the fluorescence fingerprint is not readily accessible, deconvolution can be accomplished using chemometric methods [45,49,50].

**Figure 4:**
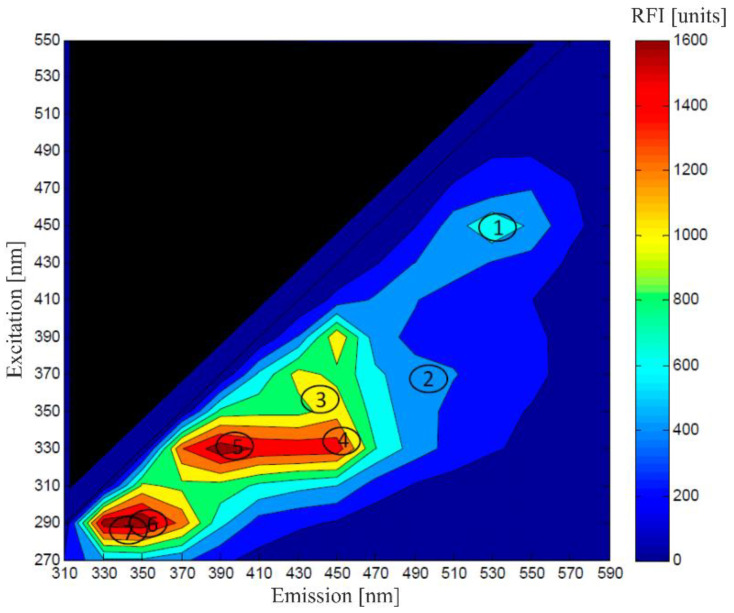
Fluorescence diagram of a fermentation broth of *S. cerevisiae* showing fluorescence signals of: (1) flavin, (2) riboflavin, (3) NADH, (4) NADPH, (5) pyridoxine, (6) tryptophan and (7) tyrosine. Adapted with permission from [47]. Faassen and Hitzmann, 2016.

The investigation of discrete emission and excitation wavelengths, particularly with respect to NAD(P)H fluorescence, has been carried out for several years [51]. Progressing instrumentation possibilities and larger capacities for data storage have enabled the detection of fluorescence fingerprints; these are created by recording the fluorescence emission spectra of several excitation wavelengths in one measuring step, producing two-dimensional fluorescence spectra, as depicted in Figure 4 [52]. Even the high-throughput application of 2D-fluorescence measurement is possible [53].

Glowacz et al. used this so-called excitation–emission matrix (EEM) spectroscopy of UV-treated and non-treated A375 cells (malignant melanoma) to determine viability by calculating the difference spectra of UV-treated and non-treated samples. The results were compared to MTT test data as a reference method and unfolded partial least squares (UPLS) regression was applied to verify whether the changes in EEMs could be related with cell viability. The proposed method achieved a high determination coefficient of R^2^ = 0.986, and thus, provides a promising basis for the development of a spectroscopic soft sensor [45]. The application of such a spectrofluorometric soft sensor in monitoring the viability (among other parameters) of industrially relevant CHO cells has been investigated in multiple research projects up to a bioreactor volume of 5000 L [49,54,55,56,57].

In recent years, many studies have been conducted in the field of monitoring cell viability in microalgae cultivation using fluorescence sensors [58]. Sá et al. measured the fluorescence EEMs of a cultivation of *Dunaliella salina* using an immersed optical fiber probe. A chemometric model was constructed by applying PCA and PLS with fluorescence data as the input and cell concentration and cell viability as outputs, which led to R^2^ values of 0.92 for cell density and 0.79 for viability [50,59].

Fluorescence microscopy can also be applied to assess cell viability based on cellular autofluorescence [60,61,62,63]. Although fluorescence microscopy only enables off-line monitoring of single cells or a distinct cell population, results might be generalized for fluorescence spectroscopy of suspension cells as well. In addition fluorescence microscopy can be adapted for on-line measurement using flow chambers or in situ microscopy [64].

Mixtures of live and dead myoblasts were analyzed by Dittmar et al. via two-photon microscopy and confocal microscopy regarding their fluorescence properties [60]. While viable cells mainly emitted blue fluorescent light with a peak intensity around 470 nm, dead cells showed a maximum fluorescence emission around 560 nm. Mainly flavoproteins contributed to emission at around 560 nm while the main fluorophore in the 470 nm range was NADH. Since relative concentrations of NADH and flavoproteins change during cell death, the fluorescence maxima of the cell change as well. One explanation for this is the arrest of the respiratory chain, which leads to the accumulation of the reduced electron source NADH inside the cell, as electrons cannot be transferred to oxygen anymore [62,63,65]. Moreover, the breakdown of mitochondrial membrane integrity exposes many proteins, leaving them unprotected from oxidation [66,67]. While flavoproteins do not show any fluorescence in their reduced form, their oxidized state is highly fluorescent, leading to increased fluorescence around 560 nm with cell death [61]. Dittmar et al. successfully distinguished living and dead cells by determining a fluorescence ratio of 475–525 nm and 560–615 nm. A threshold value of 0.68 could be set, which led to a sensitivity of 91% and a specificity of 93% [60]. Similar results were obtained in other experiments with bovine disc cells [61]. 

The fermentation of *Cupriavidus necator* has recently been monitored by Müllerová et al. wherein they also measured green autofluorescence. Flavin-related green fluorescence was analyzed using fluorescence microscopy and flow cytometry, enabling the distinguishment of live cells, dead cells and abiotic particles with high precision [68]. 

#### Time-Resolved Fluorescence Spectroscopy and Fluorescence Anisotropy

Another property which can be analyzed is the fluorescence lifetime, which enables researchers to resolve individual compounds of the complex culture broth and is able to provide detailed information about the binding state of a compound [66]. Fluorescence lifetime is defined by the average time the molecule spends in the excited state while emitting its energy as electromagnetic waves [47]. Lifetime measurements are often more prone to errors than conventional fluorescence measurements as they are more dependent on background interference, detector sensitivity and changes in turbidity. Nevertheless, time-resolved fluorescence measurements seem to be a promising tool to probe the function of the respiratory chain and are able to distinguish overlapping fluorescence signals [69,70].

In 1992, Schneckenburger et al. performed off-line time-resolved fluorescence spectroscopy of *Saccharomyces cerevisiae*, with particular attention paid to NAD(P)H and flavin fluorescence, using a charge-coupled device (CCD) camera with sub-nanosecond resolution [69]. The fluorescence decay curves of NAD(P)H (450 nm) and flavins (500 nm) showed triexponential behavior consisting of short- (0.2–0.5 ns), middle- (1.4–3.0 ns) and long-lived components (6.0–8.0 ns). While flavoproteins mainly contributed to the short-lived components, free flavin molecules may be attributed to the long-lived components. For cells with an intact respiratory chain, a lower fluorescence lifetime of the short-lived components could be observed, indicating that the ratio of free/protein-bound flavins depends on the function of the respiratory chain [69].

During cell death, the ratio of free to bound NADH changes, which results in changes in fluorescence decay time, as free NADH has a different decay time to protein-bound NADH [71,72]. Since the fluorescence decay of NADH is also influenced by the geometry of cellular membranes, fluorescence decay behavior is additionally likely to change when mitochondrial membrane integrity is disturbed due to cell death [66].

The application of fluorescence lifetime measurements to flow cytometry for the analysis of viable cells was investigated by Houston et al. [70]. Even though this is not an on-line technique, the results might be used for the implementation of further on-line analysis of cell viability based on fluorescence decay properties.

However, both fluorescence spectroscopy and fluorescence lifetime measurements also have disadvantages that become particularly apparent if the discrimination of viable and dead cells is based on NAD(P)H fluorescence. Fluorescence spectroscopic techniques only offer limited possibilities to distinguish between protein-bound and free NAD(P)H. Despite the binding of NAD(P)H to proteins shifting the fluorescence emission maximum up to 20 nm, this shift is comparatively small compared to the total width of the NAD(P)H spectrum, often allowing no clear resolution of the spectra regarding the state of the present NADH [73,74]. Fluorescence lifetime measurements are somewhat more sensitive, as the fluorescence lifetime increases up to tenfold when NADH is present in bound form [66,75]. However, short-lived components of the multiexponential fluorescence decay often resemble the decay time of free NADH [73]. An alternative is time-resolved fluorescence anisotropy, as this technique can clearly distinguish between free and bound NADH [73].

Fluorescence anisotropy describes the effect in which light emitted by a fluorophore can be polarized to different degrees in the respective directions as a result of rotational diffusion. The observed anisotropy is defined as the ratio of the difference between parallel and perpendicular polarization (depending on the polarization of excitation) to the total intensity of the fluorescence in all three dimensions [73,76]. 

The binding of NADH to proteins leads to an up to tenfold enhancement of the anisotropy decay time, since free and bound NADH are extremely different in size, and thus, exhibit different rotational mobility, with protein-bound NADH being almost immobile [73]. 

Although there are no experiments investigating the fluorescence anisotropy of NADH with respect to cell viability, many studies on the functioning of the respiratory chain have been performed, including the use of inhibitors and uncouplers of the respiratory chain [73,74]. Transferability of the results to viability measurements is therefore conceivable.

The change in the fluorescence anisotropy decay time of hippocampal rat cells under normoxic and hypoxic conditions was studied by Vishwasrao et al., whereas Yuan et al. analyzed variance in fluorescence anisotropy between melanocytes and melanoma cells when the electron transport chain was blocked [73,74]. During hypoxia, the average fluorescence lifetime decreased as the concentration of the faster decay species increased. Anisotropy showed a rapid initial decay, a rise and a second slower decay, indicating a mixture of fluorophore species (associated anisotropy), e.g., bound and free NAD(P)H. Associated anisotropy results in multiexponential behavior, with hypoxia affecting the relative concentrations amongst the four species. In particular, for the three bound species, a redistribution of NADH seems to take place at the protein binding sites, whereby more NADH is bound to proteins, contributing to a shorter decay time [73]. Comparable results were also obtained when cells were treated with an inhibitor of the respiratory chain. The fluorescence anisotropy decreased when the electron transport chain was blocked by rotenone treatment, indicating an increase in the ratio of free-to-protein-bound NADH in the treated cells [74].

### 3.3. Infrared Spectroscopy

Infrared (IR) spectroscopic techniques have also been increasingly applied to assess the state of cultivated cells. Methods that utilize electromagnetic radiation with a wavelength of 740 nm to 2500 nm are referred to as near-infrared (NIR) methods, whereas mid-infrared (MIR) methods use wavelengths in the range of 2500 nm to 25,000 nm [77,78]. The far-infrared range with wavelengths above 15 µm is rarely used for bioprocess monitoring [20]. 

IR spectra are able to show alterations in the dipole moment of molecules when excited at defined frequencies that result in a change in the vibrational energy of the matter [8,26]. IR spectroscopy often produces very broad peaks, which are caused by the overlapping of signals. Additionally, for this spectroscopic method, resolving the spectra into its constituents via PCA methods is mandatory [79]. 

Spectroscopy in the NIR range of 12,500 cm^−1^ to 4000 cm^−1^ is electronic spectroscopy, as well as vibrational spectroscopy, as bands arising from electronic transitions and those produced by overtones and combinations of vibrational modes overlap [80]. MIR radiation mainly involves the production of changes in the rotational vibration of functional groups of organic compounds [77].

However, it has to be considered that the application of IR methods in biotechnology is limited due to the low penetration depth of IR radiation and the high absorbance of water above a wavelength of 2500 nm, and measurements can usually only be performed in the near- to short-wave IR range (800–2500 nm) or with a short path length [80,81]. Since flow-through cells often do not fulfill the short path length, IR spectra are mostly acquired using spectrometers combined with an immersed fiber-optic probe [82,83]. 

Additionally, many substances only have a small molar absorptivity in the NIR range, resulting in a limited application of the method to target analytes with a low concentration (e.g., key metabolites). The most specific absorption patterns arise within the so-called fingerprint region (500–1500 cm^−1^). In consequence, this MIR region is predominantly used for the identification of multiple compounds in the culture broth [13,77]. 

The measurement principle of attenuated total reflectance (ATR), which is based on the reflection of light at the phase interface of two media with different refractive indices, cannot be used to assess cellular states as the cells are too big to enter the measuring zone [20,77].

The application of IR spectroscopy to determine cell viability has been analyzed in some studies with CHO cells [84,85,86,87]. The use of IR spectroscopy for process control by determining the concentration of major metabolites is already common [83,88]. Sandor et al. studied the non-invasive monitoring of eight CHO cell cultivations via NIR and MIR spectroscopy in order to predict critical process parameters including viability. Spectral acquisition was performed in the range of 950 nm to 1650 nm for NIR and of 3000 cm^−1^ to 700 cm^−1^ for MIR and the obtained spectra were pretreated via the detrending and calculation of the first and second derivatives. The main data processing included PCA and PLS. Using NIR spectroscopy, cell density and viability could be predicted with a root mean square error of prediction (RMSEP) of 3.9 × 10^6^ cells/mL and 3.62%. With cell density being the first principal component (PC) in PCA of the NIR spectra, and the main metabolites being the second PC, cell viability was the third principal component. For the construction of a good calibration model for cell viability, four factors resulted in R^2^ = 0.922. Due to significant changes in cell texture and morphology during cell death, even direct measurement of viability via NIR spectroscopy was feasible. Additionally, it was possible to estimate cell parameters based on light-scattering effects in the NIR range [84].

The application of the simpler and less expensive MIR devices, especially for small-scale cultivations and in academia, was successfully investigated by Capito et al. in cultivations of CHO cells. Values of relative cell viability between 20% and 95% could only be predicted with an error of 8.82%. While it was not possible to set a model for determination of the number of non-viable cells, the model for monitoring the number of viable cells was better than in previous publications using MIR. If less accurate measurement results are acceptable, MIR is also suitable for determining viability in bioprocesses [86].

While a lot of studies have been conducted in applying IR spectroscopy to mammalian cell cultures, IR spectroscopy is not very popular in microbial bioprocess monitoring. Nevertheless, IR spectroscopy has been deployed in the detection, differentiation, quantification and characterization of bacteria, yeast and fungi [82,89,90,91,92]. Even though many studies do not focus on the determination of viability, the techniques and results of experiments for biomass estimation mentioned in this review might be transferable to the determination of viability.

Mostly, IR spectroscopic experiments for monitoring microbial cultivations were performed using immersion probes. Arnold et al. established a fiber-optic in situ NIR spectroscopic sensor with an adjustable path length to monitor biomass in an industrial fed-batch *E. coli* process, enabling the alteration of measurement parameters depending on total cell density [89]. In contrast, NIR reflection spectra of a cultivation of *Penicillium chrysogenum*, which were directly acquired through the glass wall of the bioreactor, were used for the prediction of biomass and penicillin concentration by Zimmerleiter et al. [82]. 

De Sousa Marques et al. showed the possibility of distinguishing *E. coli* and *Salmonella enteritidis* in pineapple pulp using NIR spectral information. In particular, a wavelength range between 1110 nm and 2000 nm combined with multivariate data analysis (PLS-DA) was well suited to distinguishing the two bacterial species [90]. The application of NIR spectroscopy in the detection of food contaminants has already been successfully realized in other studies [91].

NIR is very sensitive to changes in membrane structure due to the characteristic vibrational frequency of each functional group of membrane macromolecules. Thus, it can be assumed that, similar to a human fingerprint, each bacterial species generates unique NIR signals [91]. If it is possible to distinguish bacterial species on the basis of their different membrane compositions, then it might also be possible to distinguish living and dead cells, since these often differ fundamentally in membrane integrity [59].

Commercial probiotic food supplements containing different bacterial strains, e.g., *Lactobacillus acidophilus*, *Bifidobacterium bifidum* and *Lactococcus lactis*, have recently been characterized by Bósquez et al. with regard to the viability of the embedded bacteria [92]. Notwithstanding that only poor values have been obtained so far for the regression of colony-forming units (R^2^ = 0.82), this demonstrates the particular relevance and great potential of IR spectroscopic methods for the determination of viability in biotechnological processes.

### 3.4. Raman Spectroscopy

Raman spectroscopy is another spectroscopic method which allows sensitive, non-invasive, high-speed bioprocess monitoring. It is based on the detection of inelastic scattering of monochromatic light, which occurs when incident light interacts with sample molecules [14]. While Rayleigh scattering accounts for the majority of the scattered light, with the frequency of the scattered light not changing, a small fraction undergoes a shift in the original wavelength. This is called Raman scattering [14,28]. The shift in wavelength depends on the chemical bonds present in the analyte molecules, and the detected signal can provide information on both the vibrational and rotational characteristics of the target molecules [14,28]. Thus, like infrared spectroscopy, Raman spectroscopy is a vibrational spectroscopy, but is based on light scattering. In contrast to NIR and MIR spectroscopy, polar molecules such as water show weaker absorption, which makes this technique very convenient for qualitative and quantitative analysis of aqueous culture broth [8]. 

However, relatively high analyte concentrations are required to obtain a detectable scattered light signal, since the molecules’ probability of undergoing a Raman state transition is very low. In addition, in some wavelength ranges, Raman signals may be overlapped by fluorescence signals, which are often much more intensive [8,26]. 

For bioprocess monitoring up to the industrial scale, Raman spectroscopy is mainly applied via optical fibers [14]. As long as the reactor is equipped with a glass window for 180° scattered light measurement, Raman spectroscopy could also be applied to single-use bioreactors [20].

Since the development of adjustable lasers has advanced substantially, Raman spectroscopy is increasingly used for monitoring metabolites, and total and viable cell density in microbial cultivation and in mammalian cell culture. However, chemometric techniques for data acquisition are always essential for spectra evaluation, and thus, are firmly linked to Raman spectroscopy [1,2,13,14].

In the literature, Raman spectroscopy has been most frequently used in the cultivation of CHO cells, which are the most widely used host for the industrial production of biopharmaceuticals [81,93,94]. 

Process variables which have been regularly monitored in situ via Raman spectroscopy are glucose, glutamine, lactate, ammonia, total cell density and viable cell density [81,95,96]. In most of the studies, the acquisition of relevant information from (preprocessed) spectra was achieved via PLS regression (PLSR) [93,95]. The transferability of the Raman spectroscopic methods to a larger, industrial scale was tested multiple times. The standard errors for the individual process parameters were largely of the same order of magnitude as comparable off-line regression methods [93,94,95]. 

Recently, a combination of off-line NIR Raman spectroscopy and MVA was used by Novikova et al. for the classification and viability assessment of phytoplankton in marine environments [97]. Here, Raman spectroscopy enabled the differentiation of phytoplankton species even when the pigment profiles were very similar amongst the species to be distinguished. Additionally, viable and non-viable cells were successfully classified, with sensitivities and specificities ≥95%, using a two-component PLS-DA model [97]. The use of Raman spectroscopy to differentiate viable and non-viable phytoplankton has also been tested on UV-treated ballast water [98].

A practical application of high-content analysis Raman spectroscopy (HCA-RS) for viability testing was developed by Mondol et al., enabling the analysis of a high number of samples without any user intervention. PCA combined with a support vector machine (SVM) was used to predict the viability of a mixed cell population treated with doxorubicin with high accuracy [99].

Since it was first applied to industrial bioprocesses in 2011, Raman spectroscopy is increasingly used for process monitoring and control. In addition to relevant metabolite concentrations, cell viability can be predicted with high accuracy [100,101].

### 3.5. Dielectric Spectroscopy/Capacitance Sensors

**Figure 5:**
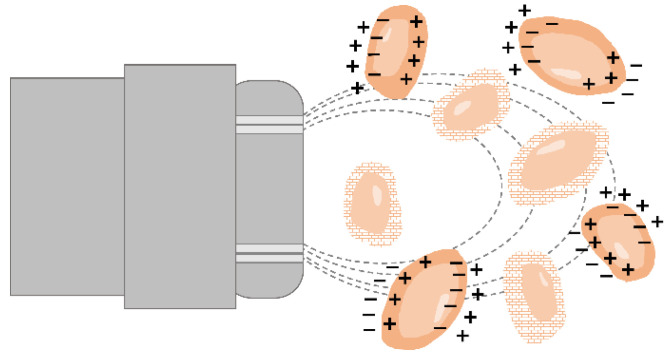
Principle of capacitance measurement for cell viability determination. Only viable cells are polarized by the electric field and contribute to the capacitance signal. Dead cells cannot be polarized.

The dielectric properties of cells in a conductive medium can be used for cell viability estimation, as well [102]. Whereas the lipid layer encapsulating the cells is nonconductive, the cytoplasm is a highly conductive medium containing salts, water, proteins and nucleic acids. Thus, cellular polarization occurs when an electric field is applied to a suspension of cells as the cellular ions move towards the electrode with an opposite charge but are stopped by the cell membrane [103]. Hence, cells with an intact membrane exhibit capacitance behavior, and consequently, permittivity values can be measured, while cells with a disrupted membrane cannot be polarized (Figure 5) [1,13,14]. The overall capacitance of the culture broth correlates with the number of viable cells as only cells with intact membranes behave as capacitors [8,13,104]. The capacitance (measured in picofarad pF) depends on the degree of polarization, and the overall polarizability of the cell suspension corresponds to the permittivity (pF per cm) [1]. 

It has to be taken into consideration that due to the non-uniform cell size, measurement of capacitance (radio-frequency impedance; dielectric spectroscopy) is not a determination of the number of viable cells, but rather, a measurement of viable cell volume. Consequently, for the estimation of viable cell concentration, it has to be assumed that all cells are the same size, which is often not the case during the stationary and decline phases [14,105,106]. Thus, linear calibration models usually cannot sufficiently describe the relationship between viability and capacitance, especially during the later phases of cell growth. Multivariate models such as PLS modeling or Cole–Cole models are often more appropriate to predict viability during the entire cultivation time [14]. 

Since cell death is usually accompanied by a loss of membrane integrity, measurement of capacitance is not suitable for determining total cell count. Therefore, capacitance sensors are often combined with turbidity sensors [1,103,107]. 

For many years, monitoring of viability in industrial bioprocesses has been carried out based on capacitance measurement. Capacitance probes fitting common bioreactor ports are commercially available from several companies [102]. Some well-known sensors include the FUTURA® biomass probe (Aber Instruments Ltd., Aberystwyth, UK), the Incyte sensor (Hamilton Bonaduz AG, Bonaduz, Switzerland) and the BioPAT® ViaMass probe (Sartorius Stedim Biotech, Göttingen, Germany) [5,6,7].

The majority of the research work on the development and testing of capacitance sensors has been conducted with CHO cultivations [105,106,108,109,110]. However, the monitoring of other mammalian cell lines and various prokaryotic and eukaryotic microbial cultivations via capacitance measurement has also been widely studied [111,112,113,114,115]. Special emphasis has also been placed on brewery yeast management in order to avoid over-pitching and improve the brewing process [104].

The application of an on-line capacitance probe to monitor the viable cell concentration during the cultivation of CHO cells combined with multivariate data analysis was investigated by Metze et al. [108]. In this study, the use of frequency scanning instead of single frequencies provided reliable information about viable cell density and could cope with changing cell diameters during the cultivation process. A multivariate model trained with data from five standard cultivations in a small-scale single-use bioreactor enabled the prediction of viability, with errors between 5.5% and 11%; it was then applied to process deviations such as dilution steps or feed variations to test its robustness [108]. It was shown that capacitance measurements can be applied to overcome the overestimation of viability from regularly occurring when trypan blue staining is performed, since trypan blue staining cannot recognize apoptotic cells with an intact membrane [105]. 

Experiments on a commercial fed-batch process with CHO cells revealed a limit of quantification (LOQ) of 5 × 10^5^ viable cells per mL and also showed the scalability of capacitance measurement, as the correlation of biocapacitance and viable cell density was consistent across four orders of magnitude in terms of reactor volume [106,109].

Furthermore, capacitance measurements were used for differential analysis of the induction of cell death [116]. Lee et al. showed the feasibility of analyzing endocytosis and the screening of chemotherapeutic agents via the measurement of capacitance [116]. Investigation of the antimicrobial activity of various agents can be performed by employing capacitance measurements, as well [117]. However, results show that depending on the type of cell death or the mechanism of action of the agent used, the results of capacitance measurement do not necessarily provide reliable values for viability determination. This is especially the case when the cell membrane remains intact during cell death [117]. Deviations can also result from fluctuations in intracellular composition, e.g., lipid content [103]. Nevertheless, capacitance measurements offer the great advantage of not being influenced by abiotic particles in the medium, and thus, represent a promising spectrometric method for viability measurement in bioprocess monitoring [102].

## 4. Soft Sensors

As mentioned several times in previous examples, the determination of many target variables of a bioprocess is often not possible or straightforward. In addition to cell viability, these variables may include biomass, product concentration, substrate concentration, product quality and specific growth rate. The inability to measure these variables can be due to a variety of reasons. Sensors for their measurement do not yet exist or cannot be integrated into the process due to structural conditions or high acquisition and maintenance costs. Consequently, some process parameters can only be determined using off-line methods. However, since these methods are often time-consuming and only provide time-delayed results, at-time intervention in the process is not possible [118,119,120]. 

The solution to these problems can be found in software sensors (soft sensors) [121]. These sensors provide fast, indirect on-line monitoring of the targeted variables, and thus, enable improved control of the process [120]. The sought-after (dependent) variables are calculated by soft sensors based on measurable (independent) process variables [122]. Soft sensors are composed of a hardware and a software component. While the hardware part consists of the sensor itself, which is used in the process, an algorithm or model applied to calculate the dependent variables forms the software part [119]. For the evaluation of soft sensors, the coefficient of determination (R^2^) and the RMSE or RMSEP, the mean error calculated from the root of the mean square error between the calculated values and the actual (off-line) determined values are used [123].

Soft sensors can be divided into three categories: model-driven sensors, also known as white-box sensors; data-driven sensors, also known as black-box sensors; and a hybrid form which combines both model- and data- driven sensors, the gray-box sensors [118,119,120].

### 4.1. White-Box Sensors

White-box sensors are based on mechanistic models (first principle models) [118,119]. For white-box sensors, knowledge about the process is necessary to create mathematical models describing the process. These models are then used to calculate the dependent variables. The creation of these models can be a great challenge, since detailed knowledge about the process (chemical transformations, kinetics) is often not available [119]. Furthermore, these models are often created for the planning of process plants and represent the optimal conditions for production; thus, they can lead to deviations from the actual conditions in the process [122]. In addition, the calculation of these models is time-consuming, which can cause a delay in process control [118]. Although the monitoring of viability using white-box sensors has not been investigated to date, models created for the determination of biomass may be applicable to viability, as well, if they are adapted appropriately. One possible application of these models is the determination of the biomass of *E. coli* and *Komagataella phaffii* in a cultivation, as shown by Sagmeister et al. The biomass can be inferred by measuring the substrate consumption and the carbon balance using the established process model [124].

### 4.2. Black-Box Sensors

Black-box sensors use process data to determine the targeted variables of the process they were trained on. MVA or artificial neural networks (ANN) can be applied for this purpose. Black-box sensors do not require detailed process knowledge; hence, they are the most commonly used soft sensors. In addition, the actual state of the process is represented more accurately using black-box sensors since they process real-time data of the cultivation [118,122]. PCA and PLS(R) are the most frequently applied data processing techniques. While PCA enables the detection of correlations in the data, and thus, a dimensional reduction, PLSR analyzes the covariance between the process variables and target values (determined off-line) and allows the prediction of these target values [125]. Furthermore, SVM, ANN, neuro fuzzy systems, as well as combinations of the mentioned methods can be applied [119]. ANNs are particularly suitable for non-linear and complex problems with, e.g., similarities to kinetics [120,126].

The available input variables and their properties, the order of the procedures and the degree of non-linearity determine the choice of the data processing method. All these parameters contain information which must be taken into account and is fed into the models, and thus, evaluated. Therefore, the user must sift through the data and carefully select which data are relevant and which data hinder the calculation [120].

Besides spectroscopic data, microscope images can also be used as input data, which can be analyzed via deep learning-based image processing algorithms including convolutional neural networks [127,128,129].

In their work, Claßen et al. and Sá et al. illustrate examples of the use of black-box soft sensors to determine cell viability. Two-dimensional fluorescence spectra in combination with PCA and PLSR have been used to determine cell viability and cell concentration and to gather additional information about cell metabolism. In these examples, CHO cells and *D. salina* were cultivated. The soft sensor of Claßen et al. (R^2^ of 0.96 and RMSEP of 3.82) showed slightly better performance than that of Sá et al. (R^2^ of 0.76 and RMSEP of 9.5) [50,56].

Successful experiments to determine cell viability have also been conducted by applying MVA methods to NIR spectra. An example of this is shown in the work of Zavala-Ortiz et al., where PLSR and locally weighted regression (LWR) were compared to determine the cell viability as well as glucose, lactate, glutamine, glycosylated monoclonal antibody (mAb) and non-glycosylated mAb concentration of a CHO cell cultivation. Due to the non-linearity of the dependent and independent variables, the LWR performed better than the PLSR, with an RMSEP of 5.5 in contrast to 8.34 for the PLSR [130].

Another (more popular) application of soft sensors, besides the measurement of viability, is the measurement of biomass. Using PCA and PLSR in conjunction with fluorescence spectra, König et al. succeeded in determining the biomass of an *E. coli* cultivation. The fluorescence signals of tryptophan, NADH and FAD/FMN were measured and, with an RMSEP of 4.6, good results in the determination of biomass were achieved [46].

ANNs are suitable for the determination of biomass, as well. Using data from a cultivation of *K. phaffii* for the production of a hepatitis B vaccine, Beiroti et al. succeeded in determining the biomass and the specific growth rate of cells by measuring the CO_2_ evolution rate, ammonia consumption rate and methanol consumption rate using an ANN. The product yield could then be increased by adjusting the feeding volumes accordingly [126].

Soft sensors can also be applied to detect defects in the process, and might be applicable to detecting changes in viability, as well. An example is the distinction of cells based on their surface properties by measuring scattered light, as performed by Rajwa et al. Using SVM and measuring scattered light intensity via flow cytometry, different bacteria (*Listeria innocua*, *B. subtilis*, *E. coli* and *Enterococcus faecalis*) could be distinguished at a rate of 68–99%, which could then be applied in the detection of process contamination [131].

### 4.3. Gray-Box Sensors

Gray-Box sensors combine model-based and data-based sensors [118]. They can often achieve higher accuracy because their programming incorporates information in the form of process knowledge [120]. In their work, Ohadi et al. combined a fluorescence-based soft sensor with PLSR and dynamic mechanistic metabolic models of cells, which enabled determination of the concentration of viable cells, dead cells, recombinant proteins, glucose and ammonia [132].

Gray- and black-box soft sensors are particularly suitable for industrial applications, since they not only rely on mechanistic models but also use a large amount of data from past processes, which are usually available [120].

Some important steps have to be considered for the programming of soft sensors. A test plan according to the QbD principle and targeted data generation is recommended in order to generate usable data for the soft sensor. For soft sensors or MVA, the PAT initiative of the FDA also provides a guideline for the generation and evaluation of process-related data [125]. A variety of programs exist which facilitate MVA, as well as the programming of ANNs, for the evaluation and review of data; these include: The Unscrambler (Aspen Technology, Inc., Bedford, MA, USA), SIMCA (Sartorius AG, Gottingen, Germany), R (Free Software, version 4.2.2), Matlab (The MathWorks, Inc., Natick, MA, USA), Jmp (SAS Institute, Cary, NC, USA), SAS (SAS Institute, Cary, NC, USA) and IBM SPSS Statistics (IBM, Armonk, NY, USA) [46,50,56,123,126,130,131].

Achieving high accuracy, reproducibility, selectivity, sensitivity, robustness and stability remains the main challenge for soft sensors to provide a reliable replacement for off-line methods. Hence, the validation of soft sensors is of high importance [133]. Easier maintenance and lower acquisition costs are the main advantages of soft sensors compared to special sensors. Furthermore they are immune to mechanical faults [122]. In addition, soft sensors enable errors such as sensor failure to be detected and even intercepted in process control. Coupling to new sensor hardware and adaption to changed process conditions can be achieved with little effort [120]. In this way, soft sensors enable a more stable process, and also contribute significantly to cost savings, increase production and product quality and facilitate compliance with production specifications [118,120,134].

## 5. Conclusions and Future Perspectives

Viability sensors combine the two trends of biologization and digitalization. On the one hand, they enable the optimization and performance improvement of biotechnological processes by realizing the on-line process monitoring of cell viability. Thanks to improved process understanding, resource-conserving biotechnological production processes can be run more economically, allowing chemical production processes, which are often detrimental to the environment, to be gradually superseded by sustainable biotechnological processes. On the other hand, well-established but manual standard methods for viability determination can be replaced by digital on-line methods, so that one more step towards industry 4.0 can be taken [135,136,137]. In the future, manual process control will be replaced by automatic process operation based on sensor information.

Because they can provide small-step, real-time readouts of the current state of a cultivation, viability sensors will also provide a deeper understanding of the phases of cell growth and cell death; moreover, anomalies in the progress of the biotechnological production process, such as the arrest of cell growth due to contamination, can be detected at an early stage.

Especially in combination with chemometric methods and MVA, spectroscopic viability sensors have recently become powerful, robust and versatile tools for process monitoring, justifying the amount of research work ongoing in their field. In addition, besides viability, other parameters including total cell count and the concentration of different metabolites can be determined simultaneously using the same measurement setup. 

A further advantage would be that, in the spirit of the FAIR data principle, data from on-line viability sensors can provide rich meta-data. This set of data could be made available globally, and could then also be used for bioprocess simulation. Consequently, resources which would otherwise be consumed for process optimization could be saved [138].

Many of the spectroscopic methods presented in this review which have so far only been carried out off-line will presumably be available for on-line usage in the near future, once the hardware and software requirements are met. 

In addition to their integration in conventional bioreactors, the integration of optical sensors in single-use systems will be of increasing interest in the coming years [18]. In fact, by using fiber-optic light guides in combination with glass measurement windows or by using in situ probes, coupling to almost any bioreactor for continuous measurement can be achieved with low signal loss. Since only an appropriate optical interface needs to be available, the application of spectroscopic sensors in individual, custom-built bioreactors can be realized with little effort, supporting the future development of reactor prototypes.

In addition to the optimization of the technical properties of the sensors, improvement in the evaluating algorithms will also contribute to the further spread of optical sensors for viability determination.

Thus, with appropriate further development, many more highly sensitive possibilities for monitoring cell viability in bioprocesses will emerge. Hence, the time-consuming and costly implementation of new sensor technologies to approved bioprocesses will turn out to be profitable.

Since the majority of the sensors and spectroscopic methods listed in this work already showed good results in upscaling experiments, industrial-scale application could also be successfully accomplished in the near future, if it has not already happened. 

## Abbreviations

2D	two-dimensional
7-AAD	7-aminoactinomycin D
ANN	artificial neural networks
ATP	adenosine triphosphate
ATR	attenuated total reflectance
*B. subtilis*	*Bacillus subtilis*
calcein AM	calcein acetoxymethyl
CCD	charge-coupled device
CHO	Chinese hamster ovary
CPP	critical process parameters
CSTR	continuous stirred tank reactor
DA	data analysis
DLS	dynamic light scattering
*D. salina*	*Dunaliella salina*
*E. coli*	*Escherichia coli*
EEM	excitation–emission matrix
FAD	flavin adenine dinucleotide
FDA	food and drug administration
FIA	flow injection analysis
FMN	flavin mononucleotide
FSC	forward scatter
HCA-RS	high-content analysis Raman spectroscopy
IR	infrared
*K. phaffii*	*Komagataella phaffii*
LOQ	limit of quantification
LWR	locally weighted regression
MCR	multivariate curve resolution
MCR-ALS	multiple curve resolution–alternating least squares
MIR	mid-infrared
MS	mass spectrometry
MTT	3-(4,5-dimethylthiazol-2-yl)-2,5-diphenyltetrazolium bromide
MVA	multivariate data analysis
NADH, NADPH	nicotinamide adenine dinucleotide (phosphate)
NIR	near-infrared
OD	optical density
PAT	process analytical technology
PC	principal component
PCA	principal component analysis
PFR	plug flow reactor
PLSR	partial least square regression
QbD	quality by design
RMSE(P)	root mean square error (of prediction)
SOP	standard operating procedure
SSC	side scatter
STR	stirred tank reactor
SU	single-use
SVM	support vector machine
UPLS	unfolded partial least squares
UV	ultraviolet
UV-vis	Ultraviolet–visible range of light
